# Comparative Effectiveness of Antihypertensive Agents vs. Control in the Prevention and Management of Preeclampsia: A Network Meta-Analysis

**DOI:** 10.7759/cureus.109602

**Published:** 2026-05-25

**Authors:** Ali S Metwaly, Manhal Idris, Reham O AlSubhi, Hala Shaaban, Fatima Elzahra S Ahmed, Reem A Khawaji, Ghadeer J Zakrait, Sahar A Albehairi, Hanouf B Hosioi, Hadil E Salem

**Affiliations:** 1 Precision Medicine, Faculty of Pharmacy, Alexandria University, Alexandria, EGY; 2 Obstetrics and Gynecology, King Fahad Hospital, Al-Baha, SAU; 3 College of Medicine, University of Jeddah, Jeddah, SAU; 4 Medicine, Ibn Sina National College for Medical Studies, Jeddah, SAU; 5 Obstetrics and Gynecology, Faculty of Medicine, University of Khartoum, Khartoum, SDN; 6 College of Medicine and Surgery, Jazan University, Jazan, SAU; 7 Medicine, Jordan University of Science and Technology, Irbid, JOR; 8 College of Medicine, Almaarefa University, Riyadh, SAU; 9 College of Medicine and Surgery, Almaarefa University, Riyadh, SAU

**Keywords:** antihypertensive agents, labetalol, methyldopa, neonatal intensive care unit, network meta-analysis, nifedipine, preeclampsia

## Abstract

Hypertensive disorders of pregnancy, particularly preeclampsia, are a leading cause of maternal and perinatal morbidity. While antihypertensive therapy is recommended, clinical uncertainty persists regarding the optimal agent to maximize maternal safety while minimizing adverse neonatal outcomes such as neonatal intensive care unit (NICU) admission. This review aimed to evaluate the comparative effectiveness and safety of pharmacological antihypertensive agents versus standard care or placebo in the management of hypertensive disorders of pregnancy through a network meta-analysis (NMA). A systematic review and frequentist random-effects NMA of randomized controlled trials (RCTs) was conducted. Major databases were searched for RCTs comparing antihypertensive agents (labetalol, nifedipine, methyldopa, hydralazine, prazosin) and controls. The primary neonatal outcome evaluated in this network was NICU admission. Risk of bias was assessed using the Cochrane RoB 2 tool, and the certainty of evidence was evaluated using the GRADE framework. Twenty-five RCTs were included in the qualitative and quantitative synthesis. The network geometry was anchored by labetalol and nifedipine. In the NMA, no individual antihypertensive agent demonstrated a statistically significant reduction in the risk of NICU admission compared to labetalol. However, probabilistic ranking (P-scores) identified methyldopa (P-score = 0.859) and hydralazine (0.791) as the most favourable interventions for minimizing NICU admissions. Nifedipine showed a slightly elevated, though non-significant, risk profile compared to labetalol (RR 1.09, 95% CI 0.80-1.49). The placebo and standard care ranked lowest. The overall certainty of evidence ranged from moderate to very low due to imprecision and indirectness. Active pharmacological management of hypertension in pregnancy is superior to placebo or standard care. While labetalol and nifedipine remain mainstays for acute blood pressure control, methyldopa and hydralazine probabilistically rank higher for minimizing NICU admissions. These findings support individualized antihypertensive selection based on clinical acuity and maternal-fetal hemodynamics.

## Introduction and background

Hypertensive disorders of pregnancy (HDP), including chronic hypertension, gestational hypertension, and preeclampsia, are leading global causes of maternal and perinatal morbidity and mortality [1]. Preeclampsia, a multisystem progressive disorder clinically characterized by new-onset hypertension accompanied by proteinuria or maternal end-organ dysfunction, complicates approximately 2%-8% of pregnancies worldwide [2]. The clinical trajectory of preeclampsia carries devastating risks, including eclampsia, placental abruption, fetal growth restriction (FGR), iatrogenic preterm birth, and long-term maternal cardiovascular disease [3,4]. The prevention of preeclampsia and the acute management of hypertensive crises remain central priorities in modern obstetric care.

The cornerstone of pharmacological management of HDP is the timely administration of antihypertensive agents to mitigate severe maternal hypertension, thereby averting acute cerebrovascular and cardiovascular catastrophes while safely prolonging gestation [5]. Major international guidelines recommend oral formulations, such as labetalol, nifedipine, and methyldopa, alongside intravenous options, as first-line therapies [5,6]. Furthermore, landmark data, notably from the Chronic Hypertension and Pregnancy (CHAP) trial, have catalyzed a paradigm shift toward tighter blood pressure control (target <140/90 mmHg) in mild-to-moderate hypertension. Tight control has been shown to reduce the composite risk of preeclampsia with severe features and medically indicated preterm birth without increasing the risk of small-for-gestational-age (SGA) infants [3,7].

Despite the established imperative to control blood pressure, clinical uncertainty persists regarding the optimal selection of antihypertensive agents for both the prophylaxis and acute management of preeclampsia [8]. Traditional pairwise meta-analyses and Cochrane reviews have established that antihypertensive therapy effectively halves the risk of severe hypertension compared to placebo or no therapy; however, these studies often lack the statistical power to delineate the superiority of one agent over another concerning critical maternal and neonatal outcomes [2,9]. Recent network meta-analyses (NMAs) have attempted to bridge this gap but have yielded conflicting or narrowly focused results, as while some NMAs suggest labetalol may offer a modest benefit in reducing the incidence of preeclampsia and preterm birth [6,9], others highlight nifedipine's robust efficacy in preventing severe hypertension [8,10]. The literature flags specific beta-blockers, such as atenolol, for their association with adverse fetal hemodynamics and SGA [8,11]. Moreover, existing evidence syntheses exhibit considerable treatment heterogeneity, are restricted to isolated clinical scenarios (e.g., chronic hypertension alone), or suffer from a paucity of direct head-to-head randomized controlled trials (RCTs) [6,8,9].

There is a need for an updated synthesis that evaluates the entire spectrum of available antihypertensive agents across diverse hypertensive pregnancy profiles; therefore, this systematic review and network meta-analysis aims to evaluate the comparative effectiveness and safety of pharmacological antihypertensive agents versus control (placebo, no intervention, or standard care) in the prevention and management of preeclampsia. By leveraging both direct and indirect evidence to rank these interventions across a broad array of maternal and fetal endpoints, this study seeks to resolve persisting clinical ambiguities and provide evidence-based guidance for optimizing maternal-fetal outcomes.

## Review

Methods

Protocol Registration and Reporting Standards

This systematic review and NMA were prospectively registered in the International Prospective Register of Systematic Reviews (PROSPERO; CRD420261296624). The methodology and reporting of this study adhered to the Preferred Reporting Items for Systematic Reviews and Meta-Analyses (PRISMA) 2020 guidelines [12], as well as the PRISMA extension statement for reporting systematic reviews incorporating network meta-analyses (PRISMA-NMA) [13].

Eligibility Criteria

Study eligibility was formulated using the Population, Intervention, Comparator, Outcomes, and Study design (PICOS) framework. The population included pregnant women (aged ≥18 years) with a confirmed diagnosis of chronic hypertension, gestational hypertension, or preeclampsia with or without severe features. The interventions were pharmacological antihypertensive agents administered during pregnancy for the prevention or acute management of preeclampsia (e.g., labetalol, nifedipine, methyldopa, angiotensin-converting enzyme inhibitors (ACEi), angiotensin II receptor blockers (ARBs), and thiazide-like diuretics), irrespective of the dosage or route of administration. The comparators were placebo, no intervention, usual/standard antenatal care, and alternative active antihypertensive agents.

The primary maternal outcomes included the incidence of severe hypertension and the development of preeclampsia/eclampsia, whereas the secondary outcomes included maternal and neonatal mortality, fetal growth restriction (FGR), preterm birth (<37 weeks), and treatment-emergent adverse effects. Only RCTs, including parallel-group, crossover, and cluster-randomized designs, were included. Observational studies, quasi-experimental designs, and non-human studies were excluded.

Information Sources and Search Strategy

A systematic literature search was conducted across major electronic databases, including PubMed/MEDLINE, Embase, Cochrane Central Register of Controlled Trials (CENTRAL), CINAHL, LILACS, and Scopus, from inception to the present, with no restrictions on language or publication date. The search syntax utilized a combination of Medical Subject Headings (MeSH), Emtree terms, and free-text keywords, incorporating Boolean operators (AND/OR). The search terms included "preeclampsia", "pregnancy-induced hypertension", "antihypertensive agents", "nifedipine", "labetalol", and "methyldopa". To identify grey literature and unpublished trials, clinical trial registries (ClinicalTrials.gov, WHO-ICTRP) and conference proceedings were screened manually.

Study Selection and Data Extraction

The retrieved citations were deduplicated, and two independent investigators screened the titles, abstracts, and subsequent full texts against the predefined eligibility criteria. Inter-rater reliability was quantified using Cohen’s Kappa coefficient (κ), with κ ≥0.60 deemed acceptable [14]. Data extraction was performed independently by two reviewers using a standardized, pre-piloted extraction form to capture study characteristics, patient demographics, drug dosages, and outcomes. Discrepancies at any stage were resolved by a third methodologist.

Quality Assessment and Risk of Bias

The methodological quality of the included RCTs was evaluated by two independent reviewers using the revised Cochrane Risk of Bias tool (RoB 2) [15]. The studies were assessed across five domains: randomisation process, deviations from intended interventions, missing outcome data, measurement of the outcome, and selection of the reported result. The overall risk of bias for each trial was categorised as "low", "some concerns", or "high".

Data Synthesis and Statistical Analysis

To compare multiple antihypertensive agents, a frequentist network meta-analysis was performed using R software (version 4.5.2; R Foundation for Statistical Computing, Vienna, Austria) [16], specifically utilizing the netmeta package [17]. For dichotomous outcomes, risk ratios (RR) were calculated with 95% confidence intervals (CIs). A random-effects model was employed to account for the anticipated clinical and methodological heterogeneity, estimating the between-study variance (τ2) using the restricted maximum likelihood (REML) method.

Network geometry and transitivity: Network plots were generated to visualize the geometry of the direct comparisons. The node sizes were proportional to the number of participants, and the edge thickness reflected the number of direct trials. The fundamental assumption of transitivity was clinically evaluated by comparing the distribution of potential effect modifiers (e.g., baseline blood pressure, maternal age, and gestational age) across different treatment comparisons.

Heterogeneity and inconsistency: Statistical heterogeneity was quantified using Cochran’s Q test and the I2 statistic, alongside 95% prediction intervals (PI) to estimate the anticipated treatment effects in future clinical settings [18]. To evaluate network coherence, global inconsistency was assessed using a design-by-treatment interaction model [19]. Local inconsistency, the discrepancy between direct and indirect estimates, was evaluated using the node-splitting method (separating indirect from direct evidence (SIDE) approach) [20].

Treatment ranking:* *The relative hierarchy of antihypertensive agents was probabilistically established using P-scores, a frequentist analog to the surface under the cumulative ranking curve (SUCRA). The P-scores range from 0 to 1, with a higher score signifying a greater likelihood of an intervention being the most effective [21]. The results were synthesized and cross-tabulated in league tables.

Publication bias and certainty of evidence: Small-study effects and publication bias were visually appraised using comparison-adjusted funnel plots and statistically quantified using Egger’s rank correlation test [22]. The certainty of the synthesized evidence for primary outcomes was appraised using the grading of recommendations assessment, development, and evaluation (GRADE) framework adapted for NMA, categorizing evidence quality as high, moderate, low, or very low [23].

Results

Study Selection and Inter-Rater Reliability

**Figure 1:**
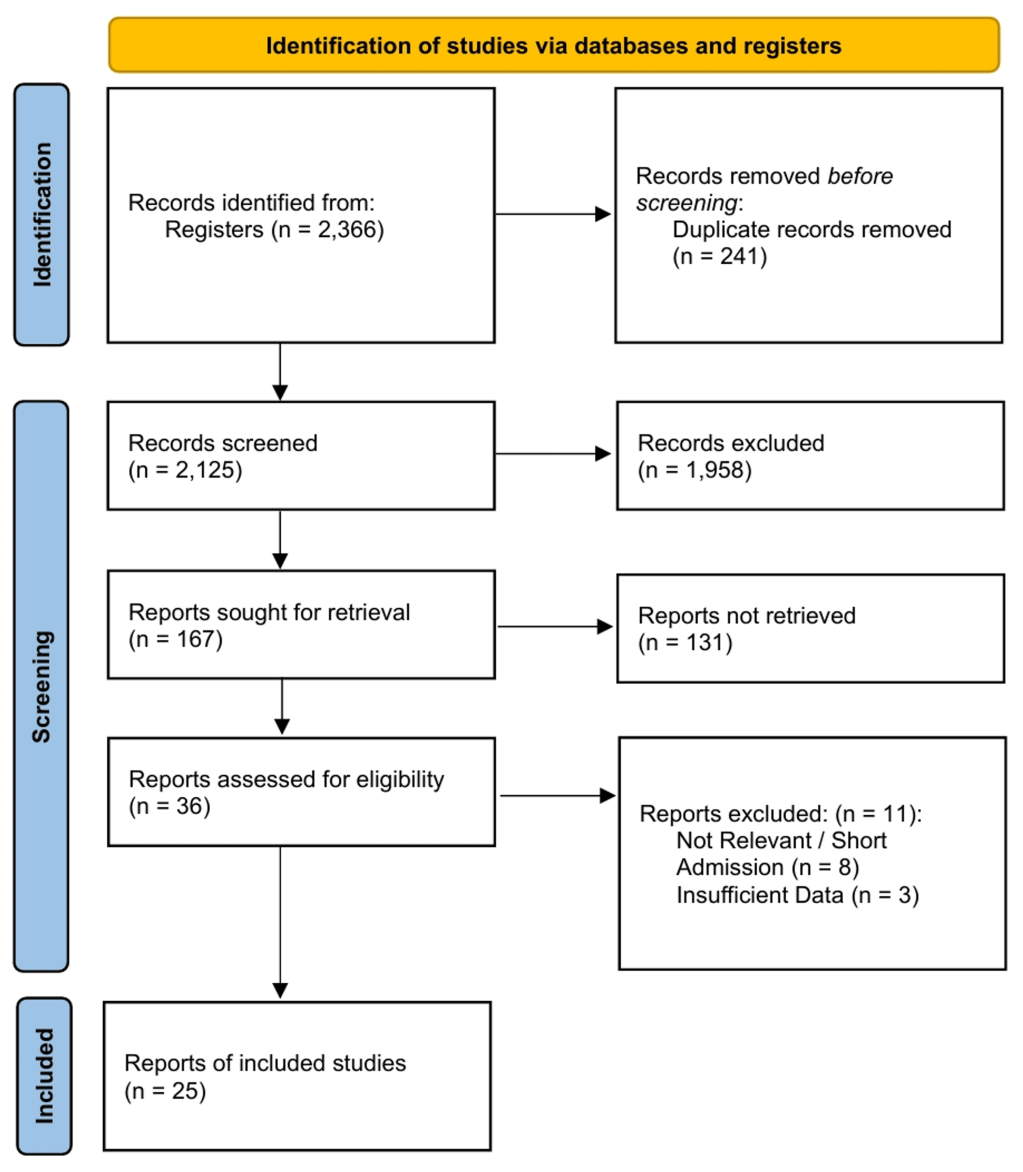
PRISMA 2020 flow diagram

The systematic literature search identified 2,366 records from the queried databases and registries. Following the removal of 241 duplicate records, 2,125 unique citations were screened at the title and abstract levels. Of these, 1,958 records were excluded because they did not meet the predefined eligibility criteria. Full-text retrieval was sought for 167 reports, of which 131 could not be retrieved or lacked sufficient data. A total of 36 full-text reports were rigorously assessed for eligibility criteria. Eleven studies were excluded (eight due to irrelevant interventions or short admission durations and three due to insufficient outcome data), leaving a final cohort of 25 RCTs for qualitative and quantitative synthesis. The inter-rater reliability during the study selection process demonstrated almost perfect agreement between the two independent reviewers (Cohen’s κ = 0.958, z = 6.78, p < 0.001). The complete study selection process is illustrated in the PRISMA 2020 flowchart (Figure 1).

Study Characteristics

The 25 included RCTs evaluated a diverse array of pharmacological interventions for the management of hypertensive disorders in pregnancy, including labetalol, nifedipine, hydralazine, methyldopa, prazosin, standard care, and placebo [24-48]. The clinical indications ranged from mild chronic hypertension to acute severe preeclampsia and hypertensive emergencies. The mean maternal age across the included trials ranged from 23.4 to 35.1 years, and the mean gestational age at enrolment varied from early pregnancy (e.g., 8.1 weeks) to late third trimester (>37 weeks). Sample sizes ranged widely, from small pilot studies (n=12) [36] to large multicenter international trials (n=2,408) [41,42]. A detailed summary of the baseline characteristics of the included studies is presented in Table 1.

**Table 1:** Baseline characteristics of the included randomized controlled trials NR = not clearly reported

Study ID	Author, Year	Population / Condition	Interventions & Sample Size (N)	Mean Age (Years)	Mean Gestational Age
[24]	Pickles et al., 1992	Mild-to-moderate pregnancy-induced hypertension	Labetalol (70); Placebo (74)	25.8 / 24.7	34.0 / 34.2 wks
[25]	Rezaei et al., 2011	Severe preeclampsia/hypertensive crisis	Nifedipine (25); Hydralazine (25)	29.4 / 29.6	35.6 / 34.2 wks
[26]	Adebayo et al., 2020	Severe hypertension (≥160/110)	Nifedipine (34); Hydralazine (35)	24.4 / 24.6	36.2 / 37.0 wks
[27]	Sharma et al., 2017	Acute hypertensive emergency	Nifedipine (30); Hydralazine (30)	23.4 / 24.2	258 / 257 days
[28]	Magee et al., 2015	Non-severe hypertension (CHIPS secondary)	Labetalol (237); Methyldopa (241)	34.1 / 35.1	~36.8 wks
[29]	Azmat et al., 2018	Acute severe hypertension	Labetalol (60); Nifedipine (60)	28.5 / 27.8	32.1 / 31.8 wks
[30]	Raja et al., 2025	Acute severe hypertension/Eclampsia	Labetalol (75); Nifedipine (75)	27.0 / 28.0	>32 wks
[31]	Sibai et al., 1990	Mild chronic hypertension (6-13 wks)	Methyldopa (88); Labetalol (86); None (90)	30.9 / 28.9 / 29.0	11.2 wks
[32]	Shekhar et al., 2013	Hypertensive emergency	Labetalol (30); Nifedipine (30)	25.9 / 26.2	36.1 / 37.3 wks
[33]	Vermillion et al., 1999	Hypertensive emergencies	Nifedipine (25); Labetalol (25)	27.2 / 27.0	34.3 / 33.6 wks
[34]	Raheem et al., 2012	Hypertensive emergencies	Nifedipine (25); Labetalol (25)	31.4 / 32.2	37.1 / 37.9 wks
[35]	Singhal et al., 2015	Mild preeclampsia	Labetalol (50); Methyldopa (50)	NR	20-40 wks
[36]	Scardo et al., 1999	Preeclamptic hypertensive emergencies	Nifedipine (6); Labetalol (6)	29.8 / 28.8	>24 wks
[37]	Hall et al., 2000	Early severe hypertension (second-line)	Nifedipine (74); Prazosin (71)	NR	28.0 / 27.0 wks
[38]	Aali et al., 2002	Severe preeclampsia	Nifedipine (65); Hydralazine (61)	27.1 / 26.8	37.0 / 37.7 wks
[39]	Salama et al., 2019	Mild to moderate chronic hypertension	Methyldopa (166); Nifedipine (160); Control (164)	NR	8.2 / 8.1 / 8.2 wks
[40]	Cleary et al., 2023	Preeclampsia (severe features, intrapartum)	Nifedipine (53); Placebo (49)	29.0 / 31.0	36.4 / 36.4 wks
[41]	Tita et al., 2022	Mild chronic hypertension (CHAP Trial)	Active Treatment (1208); Control (1200)	32.3 / 32.3	<23 wks
[42]	Sanusi et al., 2024	Mild chronic HTN (CHAP secondary)	Labetalol (720); Nifedipine (417); Standard Care (1155)	32.4 / 32.2 / 32.4	10.3 / 10.3 / 10.7 wks
[43]	Easterling et al., 2019	Severe hypertension	Nifedipine (298); Labetalol (295); Methyldopa (301)	25.6 / 25.5 / 25.5	36.5 / 36.5 / 36.7 wks
[44]	Chethy, 2024	Hypertensive emergencies	Labetalol (52); Nifedipine (52)	24.7 / 25.0	38.0 / 37.6 wks
[45]	Kaur et al., 2024	Acute hypertensive management	Labetalol (60); Nifedipine (60)	25.8 / 24.9	36.4 / 36.1 wks
[46]	Nimbark et al., 2024	Preeclampsia	Labetalol (100); Nifedipine (100)	25.5 / 25.9	NR
[47]	Jhaveri et al., 2024	Preeclampsia	Labetalol (68); Nifedipine (68)	23.9 / 23.6	34-40 wks
[48]	Rose et al., 2019	Non-severe preeclampsia	Labetalol (50); Nifedipine (50)	NR	>20 wks

Risk of Bias and Certainty of Evidence

**Figure 2:**
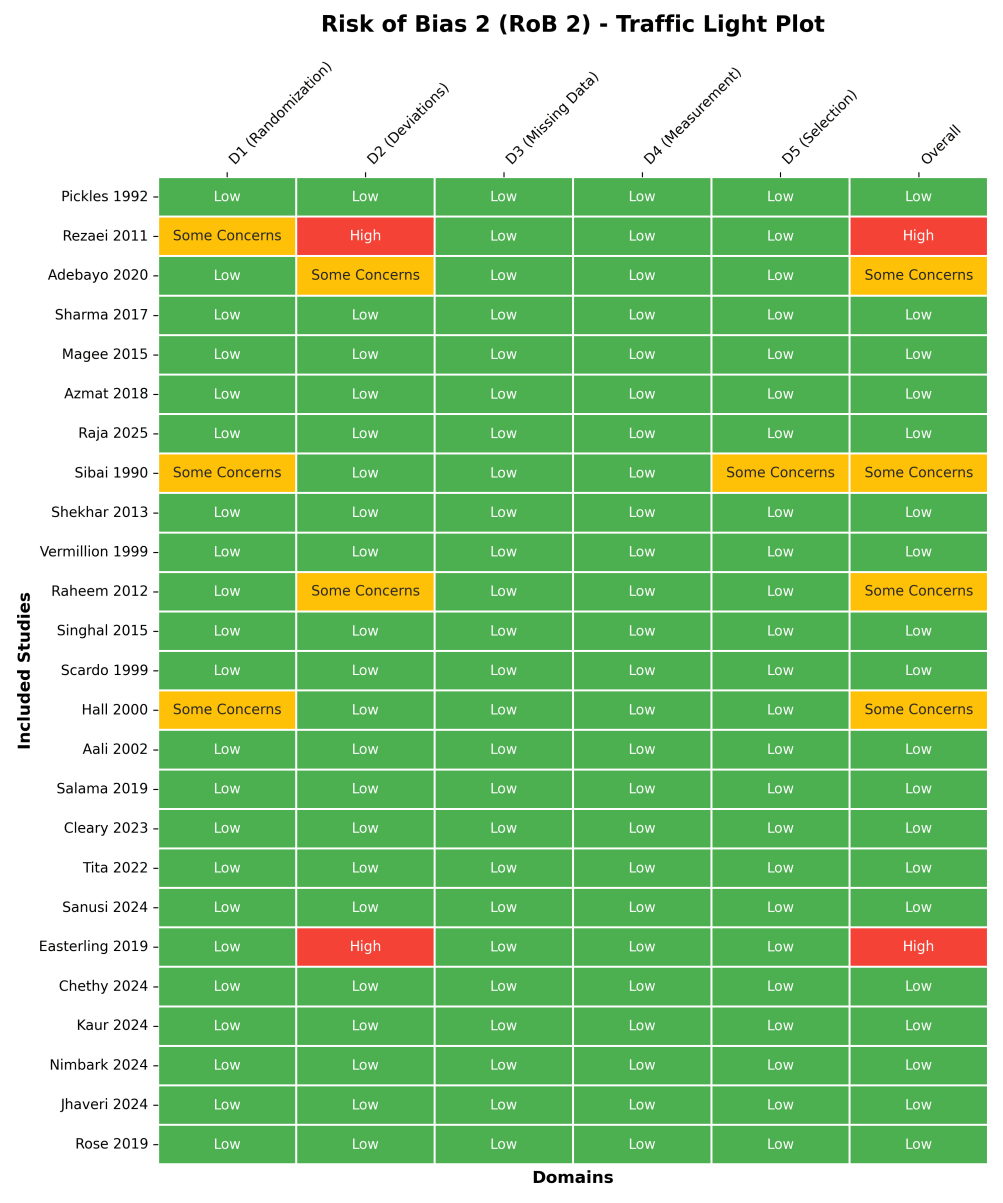
Cochrane Risk of Bias 2 (RoB 2) assessment Traffic light plot providing study-level, domain-specific quality assessments. Green indicates "Low Risk", yellow indicates "Some Concerns", and red indicates "High Risk".

**Figure 3:**
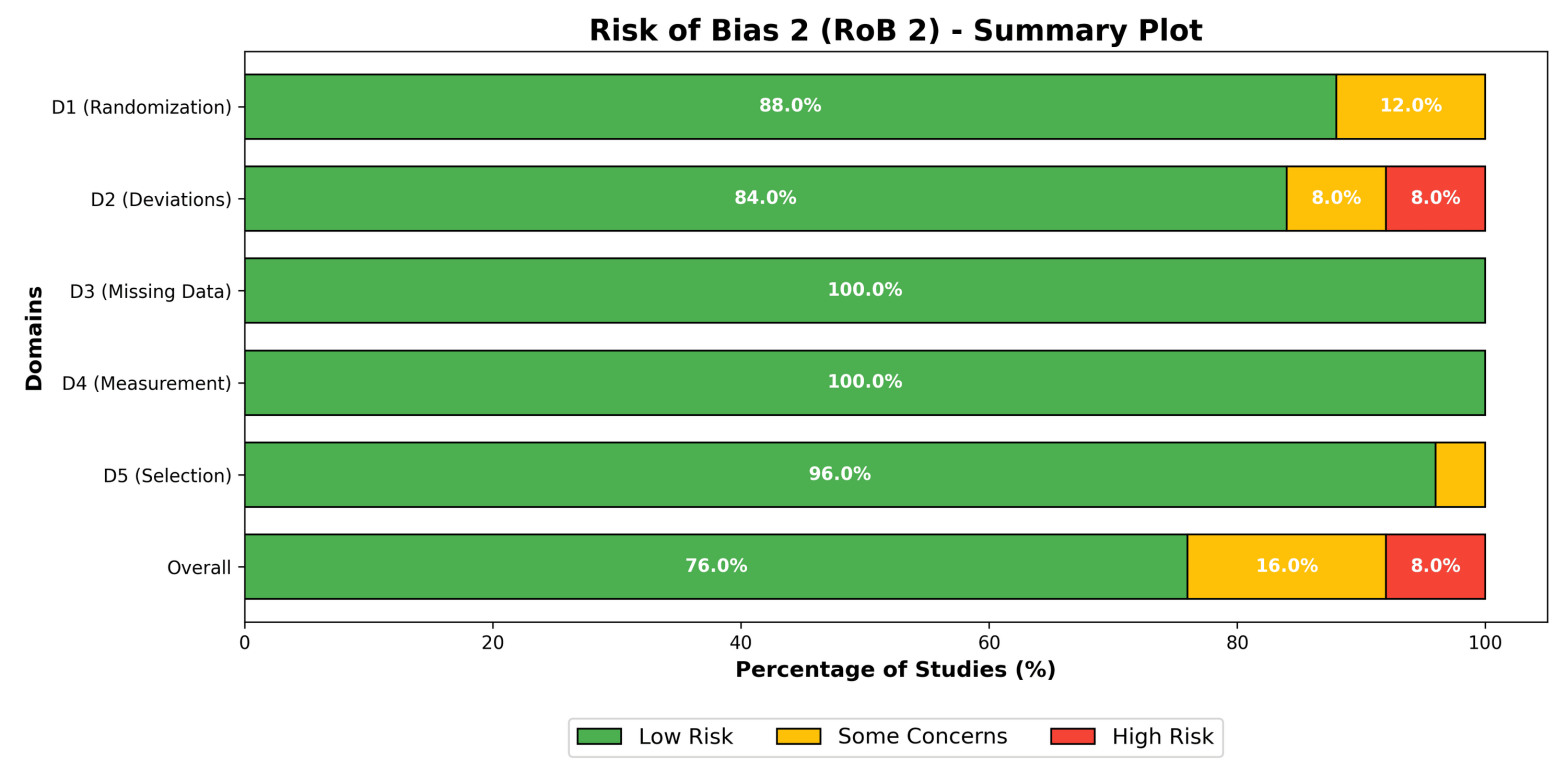
Cochrane Risk of Bias 2 (RoB 2) summary Bar plot illustrating the aggregated percentage of risk across the five evaluated methodological domains.

The methodological quality was assessed using the RoB 2 tool for RCTs. The overall risk of bias across the networks was low. Specifically, 76.0% of the included trials were rated as having a low risk of bias, 16.0% exhibited some concerns, and only 8.0% were classified as having a high risk. The primary drivers for some concerns and high-risk ratings were deviations from the intended interventions (Domain 2) due to the open-label nature of several acute-care trials and minor baseline imbalances in the randomization process (Domain 1). Visual summaries of the domain-specific evaluations are provided in the traffic light plot (Figure 2) and summary bar plot (Figure 3).

The certainty of the evidence, evaluated using the adapted GRADE framework for network meta-analysis, ranged from moderate to very low, depending on the specific treatment contrast (Table 2). Downgrades were applied due to the imprecision (wide credible/confidence intervals crossing the line of no effect) and indirectness inherent to sparse network geometries.

**Table 2:** GRADE summary of findings for a network meta-analysis GRADE: grading of recommendations assessment, development, and evaluation

Comparison (Network)	Network Estimate (RR, 95% CI)	Risk of Bias	Inconsistency	Indirectness	Imprecision	Publication Bias	Certainty of Evidence
Nifedipine vs Labetalol	0.92 (0.67 to 1.25)	Not Serious	Not Serious	Not Serious	Serious (-1)	Not Serious	Moderate
Methyldopa vs Labetalol	1.37 (0.82 to 2.29)	Serious (-1)	Not Serious	Not Serious	Serious (-1)	Not Serious	Low
Nifedipine vs Methyldopa	0.67 (0.41 to 1.08)	Serious (-1)	Not Serious	Not Serious	Not Serious	Not Serious	Moderate
Hydralazine vs Labetalol	0.67 (0.20 to 2.19)	Not Serious	Serious (-1)	Not Serious	Very Serious (-2)	Not Serious	Very Low
Hydralazine vs Nifedipine	0.61 (0.19 to 1.92)	Not Serious	Not Serious	Not Serious	Very Serious (-2)	Not Serious	Very Low

Network Geometry and Heterogeneity Assessment

**Figure 4:**
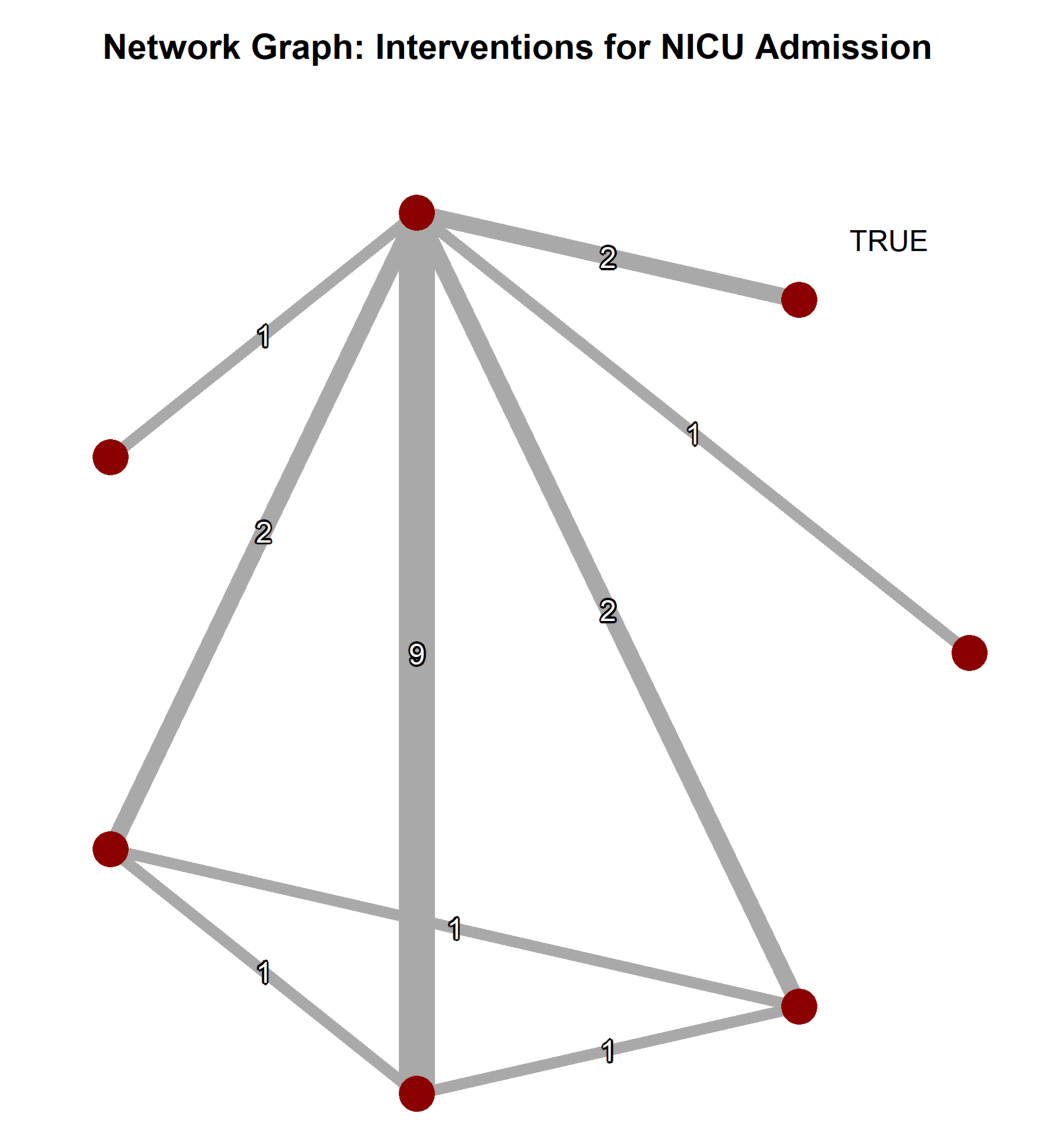
Network geometry graph for neonatal intensive care unit (NICU) admission Node sizes are proportional to the number of randomized patients, and edge thicknesses correspond to the number of direct head-to-head trials.

For the critical outcome of NICU admission, the network comprised 14 trials providing data on 4,734 observations across seven distinct treatment nodes (Figure 4). The network geometry demonstrated that Labetalol and Nifedipine nodes were the most highly connected and heavily weighted, serving as the central hubs of the network.

Statistical heterogeneity across the network was moderate, with a between-study variance (τ2) of 0.0837 and an I2 statistic of 44.17%. Cochran’s Q statistic was 19.70 (df = 11, p = 0.049). Global inconsistency was assessed using the design-by-treatment interaction (DBTI) model, which indicated the presence of statistically significant global inconsistency between designs (Q = 12.14, df = 4, p = 0.016). However, local inconsistency evaluation using the SIDE node-splitting method revealed no significant discrepancies between direct and indirect estimates for any specific comparison pair (all p-values ranging from 0.258 to 0.840), suggesting that the localized contrasts remained robust despite the global model’s variance.

Comparative Effectiveness: Network Meta-Analysis

**Figure 5:**
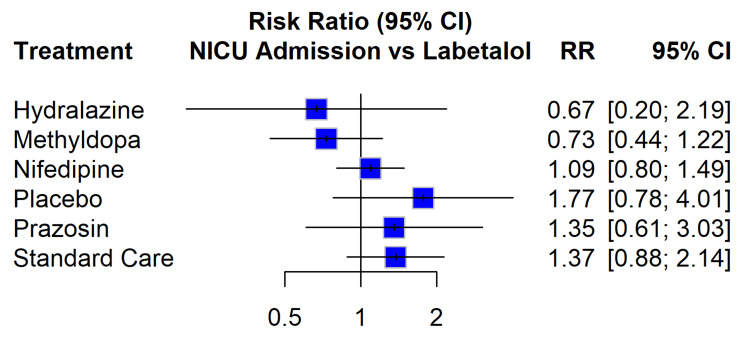
Network meta-analysis Forest plot displaying risk ratios (RR) alongside 95% confidence intervals (CI) for NICU admission, utilizing Labetalol as the central reference comparator

A frequentist random-effects NMA (REML estimator) was performed to evaluate the comparative risk of NICU admission. Using Labetalol as the central reference standard, both methyldopa (RR 0.73, 95% CI (0.44 to 1.22)) and hydralazine (RR 0.67, 95% CI (0.20 to 2.19)) demonstrated point estimates favouring reduced NICU admission rates, although these findings did not reach statistical significance. Nifedipine demonstrated a comparable, albeit slightly elevated, risk profile compared with labetalol (RR 1.09, 95% CI (0.80 to 1.49)). Predictably, the placebo (RR 1.77, 95% CI (0.78-4.01)), standard care (RR 1.37, 95% CI (0.88-2.14]), and prazosin (RR 1.35, 95% CI (0.61-3.03)) were associated with the highest risks of NICU admission relative to labetalol. The comprehensive matrix of all pairwise comparisons is presented in the network league table (Table 3), and the forest plot with 95% prediction intervals (PI) highlights the anticipated treatment effects in future settings (Figure 5).

**Table 3:** Network meta-analysis league table

Hydralazine	Labetalol	Methyldopa	Nifedipine	Placebo	Prazosin	Standard Care
Hydralazine	-	-	0.61 (0.19 to 1.92)	-	-	-
0.67 (0.20 to 2.19)	Labetalol	1.05 (0.50 to 2.21)	0.88 (0.65 to 1.21)	-	-	0.87 (0.48 to 1.56)
0.91 (0.26 to 3.17)	1.37 (0.82 to 2.29)	Methyldopa	0.65 (0.39 to 1.10)	-	-	0.45 (0.22 to 0.94)
0.61 (0.19 to 1.92)	0.92 (0.67 to 1.25)	0.67 (0.41 to 1.08)	Nifedipine	0.62 (0.29 to 1.32)	0.81 (0.38 to 1.69)	0.77 (0.49 to 1.21)
0.38 (0.10 to 1.49)	0.57 (0.25 to 1.28)	0.41 (0.17 to 1.01)	0.62 (0.29 to 1.32)	Placebo	-	-
0.49 (0.13 to 1.93)	0.74 (0.33 to 1.65)	0.54 (0.22 to 1.30)	0.81 (0.38 to 1.69)	1.30 (0.45 to 3.77)	Prazosin	-
0.49 (0.14 to 1.65)	0.73 (0.47 to 1.14)	0.53 (0.31 to 0.91)	0.80 (0.53 to 1.20)	1.29 (0.54 to 3.06)	0.99 (0.42 to 2.31)	Standard Care

Treatment ranking was established probabilistically using P-scores (the frequentist analog to SUCRA), where higher scores denoted superior clinical performance (lower risk of adverse events). Methyldopa had the highest probability of being the most effective intervention to prevent NICU admission (P-score = 0.859), followed closely by hydralazine (0.791) and labetalol (0.613). Nifedipine ranked fourth (0.501), whereas prazosin (0.326), standard care (0.260), and placebo (0.150) occupied the lowest tiers of the treatment hierarchy.

Publication Bias and Small-Study Effects

**Figure 6:**
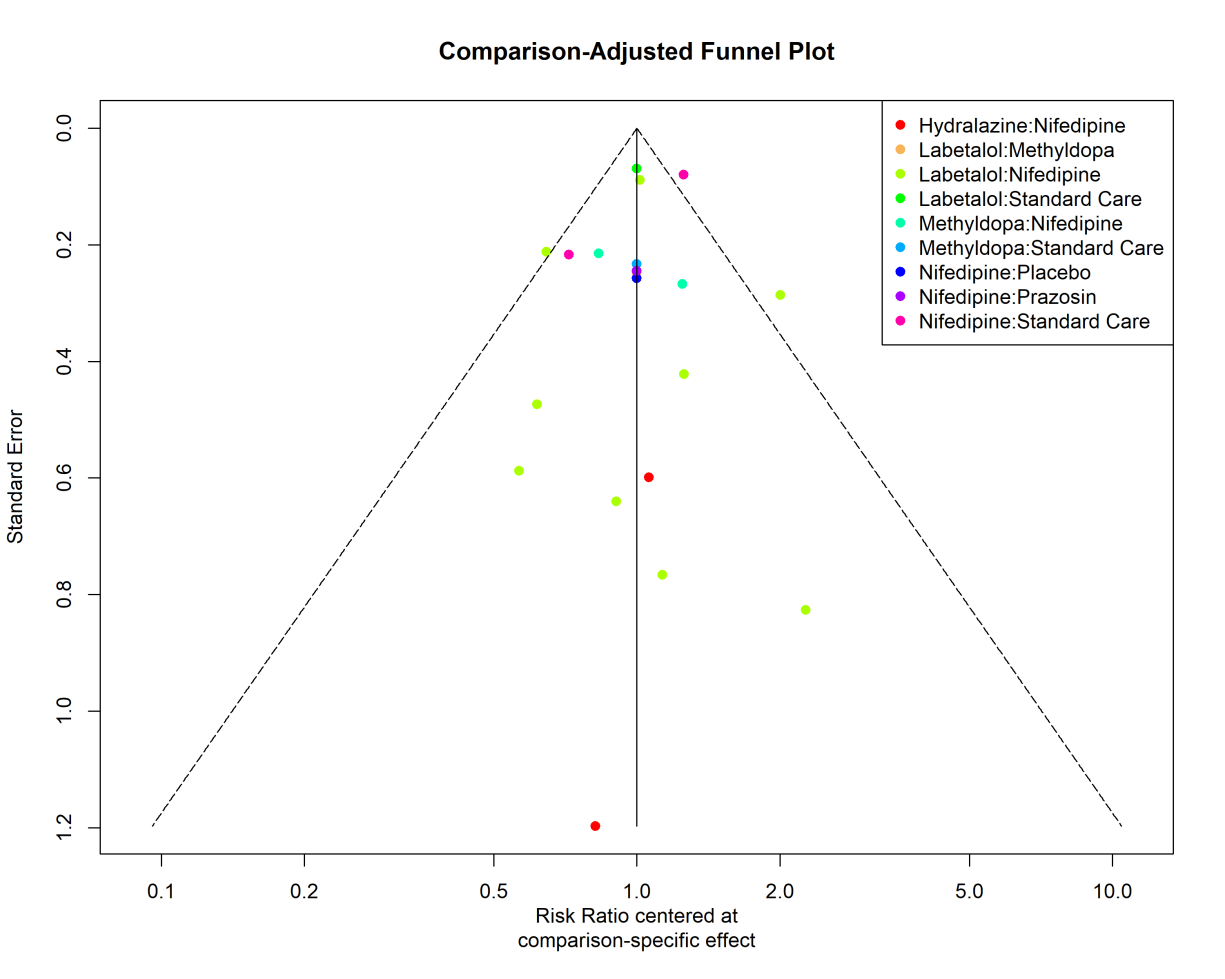
Comparison-adjusted funnel plot assessing small-study effects and publication bias across the treatment network Different colors denote distinct pairwise comparisons.

A comparison-adjusted funnel plot was generated to detect small study effects and potential publication bias (Figure 6). Visual inspection of the scatter symmetry around the zero line of the comparison-specific effect did not suggest a severe publication bias. Formal quantitative assessment via Egger’s rank correlation test (linear regression method) could not yield a valid p-value due to the sparsity of studies (k<10) per specific comparison axis; however, the lack of evident visual asymmetry corroborates the integrity of the pooled estimates.

Discussion

This systematic review and NMA synthesized evidence from 25 RCTs to evaluate the comparative effectiveness and safety of antihypertensive agents in managing hypertensive disorders during pregnancy. This network focuses on the critical neonatal outcomes of NICU admissions. The principal finding of the analysis was that while active pharmacological intervention is clearly superior to standard care or placebo, there are no statistically significant differences among the major antihypertensive agents (labetalol, nifedipine, methyldopa, and hydralazine) in terms of the risk of NICU admission. However, probabilistic treatment rankings (P-scores) suggest a hierarchy of neonatal safety. Methyldopa and hydralazine ranked highest in minimizing NICU admissions, whereas nifedipine and prazosin ranked lower. The comparison between nifedipine and labetalol, the two most utilized first-line agents, yielded an RR of 1.09 (95% CI 0.80-1.49), indicating comparable neonatal safety profiles, albeit with a slight probabilistic advantage favouring labetalol.

These findings align with and expand upon recent landmark trials and pairwise meta-analyses. The CHAP trial demonstrated that targeting a blood pressure of <140/90 mmHg reduces the incidence of adverse pregnancy outcomes without compromising fetal growth [41]. Correspondingly, this NMA ranked standard care and placebo at the bottom of the therapeutic hierarchy, reinforcing the shift toward tighter blood pressure control.

The probabilistic superiority of methyldopa in minimizing NICU admissions is a nuanced result. Methyldopa, a centrally acting α2-adrenergic agonist, has a long-established safety record in pregnancy, although its use has declined in recent years due to its slower onset of action and maternal side effects such as somnolence [28,31]. In the CHIPS trial, outcomes were broadly similar between labetalol and methyldopa, although exploratory analyses suggested that methyldopa might confer a slight protective effect against adverse perinatal outcomes in patients with pre-existing hypertension [28]. The network ranking corroborates this signal, suggesting that in non-emergent settings, methyldopa remains a highly viable and fetus-sparing therapeutic option.

Oral nifedipine and intravenous labetalol are the preferred agents for acute hypertensive emergencies because of their rapid pharmacodynamic profiles [33,40]. While some earlier observational studies and small trials raised concerns regarding nifedipine-induced maternal hypotension and subsequent fetal hypoxia [36], contemporary RCTs have largely refuted these claims [43]. This NMA confirmed that nifedipine did not significantly increase the risk of NICU admission compared with labetalol, although its lower P-score ranking suggests that clinicians should remain vigilant regarding uteroplacental perfusion when using potent peripheral vasodilators.

Clinical and Policy Implications

Current guidelines from the American College of Obstetricians and Gynecologists (ACOG) and the National Institute for Health and Care Excellence (NICE) recommend labetalol, nifedipine, or methyldopa as first-line therapies, often leaving the choice to the provider’s preference and local availability. The data support the validity of these guidelines but provide a more granular understanding of the agent-specific profiles. For acute hypertensive crises, where rapid blood pressure lowering is required to prevent maternal cerebrovascular events, nifedipine and labetalol remain the agents of choice. However, for the maintenance of chronic hypertension or stable preeclampsia, methyldopa may offer an optimized neonatal-safety profile.

Strengths

The primary strength of this study is its robust methodological framework; by utilizing a frequentist NMA, we were able to simultaneously synthesize direct and indirect evidence across all available interventions, overcoming the limitations of traditional pairwise meta-analyses. Furthermore, the inclusion of contemporary, highly powered RCTs (such as CHAP and CHIPS data) strengthens the external validity of our estimates. The rigorous application of the Cochrane RoB 2 tool and GRADE framework ensured transparent communication of the certainty of the findings.

Limitations

First, the certainty of evidence for several network nodes was downgraded to low or very low because of imprecision (wide confidence and prediction intervals) and indirectness. In addition, the included trials exhibited clinical heterogeneity; the network pooled data from both acute hypertensive emergencies (requiring intravenous interventions) and chronic maintenance therapy. While our random-effects model accounts for statistical heterogeneity (τ2 = 0.0837), the physiological responses to acute versus chronic blood pressure reduction differ and may independently influence NICU admission rates. Finally, owing to the sparsity of the network for certain contrasts, we were unable to reliably conduct meta-regression to control for gestational age at the time of intervention, which is a known independent predictor of NICU admission.

## Conclusions

In the prevention and management of preeclampsia and hypertensive disorders of pregnancy, active pharmacological treatment is imperative for optimizing maternal and neonatal outcomes. This network meta-analysis demonstrated that labetalol, nifedipine, methyldopa, and hydralazine share comparable efficacy and safety profiles regarding the risk of NICU admission. While methyldopa ranks highest for neonatal safety, the choice of antihypertensive agent should remain individualized, balancing the need for acute maternal blood pressure control against the maintenance of steady uteroplacental hemodynamics. Future large-scale, multi-arm randomized controlled trials with standardized dosing protocols are warranted to resolve the comparative superiority of these essential obstetric therapeutics.

## References

[1] Williams A, Naert M, Berhie S (2023). Preeclampsia in pregnancy: diagnosis, management, and future implications for maternal health. Contemporary Topics in Cardio-Obstetrics.

[2] Abalos E, Duley L, Steyn DW (2014). Antihypertensive drug therapy for mild to moderate hypertension during pregnancy. Cochrane Database Syst Rev.

[3] Al Khalaf S, Khashan AS, Chappell LC, O'Reilly ÉJ, McCarthy FP (2022). Role of antihypertensive treatment and blood pressure control in the occurrence of adverse pregnancy outcomes: a population-based study of linked electronic health records. Hypertension.

[4] Voto LS, Zeitune MG (2022). Perinatology. Perinatology.

[5] Ma S, Zhu L, Zhou T, Qi T, Wang W (2023). Oral nifedipine and phytosterol, intravenous nicardipine, and oral nifedipine only: Three-arm, retrospective, cohort study for management of severe preeclampsia. Open Life Sci.

[6] Hup RJ, Damen JA, Terstappen J (2025). Oral antihypertensive treatment during pregnancy: a systematic review and network meta-analysis. Am J Obstet Gynecol.

[7] Attar A, Hosseinpour A, Moghadami M (2023). The impact of antihypertensive treatment of mild to moderate hypertension during pregnancy on maternal and neonatal outcomes: an updated meta-analysis of randomized controlled trials. Clin Cardiol.

[8] Bellos I, Pergialiotis V, Papapanagiotou A, Loutradis D, Daskalakis G (2020). Comparative efficacy and safety of oral antihypertensive agents in pregnant women with chronic hypertension: a network metaanalysis. Am J Obstet Gynecol.

[9] Bone JN, Sandhu A, Abalos ED (2022). Oral antihypertensives for nonsevere pregnancy hypertension: systematic review, network meta- and trial sequential analyses. Hypertension.

[10] Govindasamy V, Kamel MA, Volucke G (2025). Efficacy and safety of nifedipine compared to intravenous hydralazine for severe hypertensive disorders in pregnancy: a systematic review and meta-analysis of randmomized controlled trials. Med Sci (Basel).

[11] Wacker J, Nwaeburu L, N'Diaye A (2024). The treatment of preeclampsia in poor and rich countries. Global Women's Health.

[12] Page MJ, McKenzie JE, Bossuyt PM (2021). The PRISMA 2020 statement: an updated guideline for reporting systematic reviews. BMJ.

[13] Hutton B, Salanti G, Caldwell DM (2015). The PRISMA extension statement for reporting of systematic reviews incorporating network meta-analyses of health care interventions: checklist and explanations. Ann Intern Med.

[14] McHugh ML (2012). Interrater reliability: the kappa statistic. Biochem Med (Zagreb).

[15] Sterne JA, Savović J, Page MJ (2019). RoB 2: a revised tool for assessing risk of bias in randomised trials. BMJ.

[16] (2026). R Core Team. R: a language and environment for statistical computing. R Foundation for Statistical Computing, Vienna, Austria. https://www.R-project.org/.

[17] Rücker G, Krahn U, König J, Efthimiou O, Schwarzer G (2023). netmeta: network meta-analysis using frequentist methods. R package version 2.8-1. https://cran.r-project.org/web/packages/netmeta/index.html.

[18] IntHout J, Ioannidis JP, Rovers MM, Goeman JJ (2016). Plea for routinely presenting prediction intervals in meta-analysis. BMJ Open.

[19] Higgins JP, Jackson D, Barrett JK, Lu G, Ades AE, White IR (2012). Consistency and inconsistency in network meta-analysis: concepts and models for multi-arm studies. Res Synth Methods.

[20] Dias S, Welton NJ, Caldwell DM, Ades AE (2010). Checking consistency in mixed treatment comparison meta-analysis. Stat Med.

[21] Rücker G, Schwarzer G (2015). Ranking treatments in frequentist network meta-analysis works without resampling methods. BMC Med Res Methodol.

[22] Chaimani A, Higgins JP, Mavridis D, Spyridonos P, Salanti G (2013). Graphical tools for network meta-analysis in STATA. PLoS One.

[23] Brignardello-Petersen R, Bonner A, Alexander PE (2018). Advances in the GRADE approach to rate the certainty in estimates from a network meta-analysis. J Clin Epidemiol.

[24] Pickles CJ, Broughton Pipkin F, Symonds EM (1992). A randomised placebo controlled trial of labetalol in the treatment of mild to moderate pregnancy induced hypertension. Br J Obstet Gynaecol.

[25] Rezaei Z, Sharbaf FR, Pourmojieb M, Youefzadeh-Fard Y, Motevalian M, Khazaeipour Z, Esmaeili S (2011). Comparison of the efficacy of nifedipine and hydralazine in hypertensive crisis in pregnancy. Acta Med Iran.

[26] Adebayo JA, Nwafor JI, Lawani LO, Esike CO, Olaleye AA, Adiele NA (2020). Efficacy of nifedipine versus hydralazine in the management of severe hypertension in pregnancy. A randomised controlled trial. Niger Postgrad Med J.

[27] Sharma C, Soni A, Gupta A, Verma A, Verma S (2017). Hydralazine vs nifedipine for acute hypertensive emergency in pregnancy: a randomized controlled trial. Am J Obstet Gynecol.

[28] Magee LA, von Dadelszen P, Singer J (2016). Do labetalol and methyldopa have different effects on pregnancy outcome? Analysis of data from the Control of Hypertension In Pregnancy Study (CHIPS) trial. BJOG.

[29] Azmat B, Sharma A, Singh P (2018). Intravenous labetalol vs oral nifedipine in acute severe hypertension of pregnancy - a randomized controlled trial. J Cardiovasc Dis Res.

[30] Raja GPT, Patil KP, Metgud MC, Savanur M, Malapure A, Hanji V (2025). Intravenous labetalol vs oral nifedipine in acute severe hypertension in pregnancy: a randomized controlled trial. J South Asian Feder Obst Gynae.

[31] Sibai BM, Mabie WC, Shamsa F, Villar MA, Anderson GD (1990). A comparison of no medication versus methyldopa or labetalol in chronic hypertension during pregnancy. Am J Obstet Gynecol.

[32] Shekhar S, Sharma C, Thakur S, Verma S (2013). Oral nifedipine or intravenous labetalol for hypertensive emergency in pregnancy: a randomized controlled trial. Obstet Gynecol.

[33] Vermillion ST, Scardo JA, Newman RB, Chauhan SP (1999). A randomized, double-blind trial of oral nifedipine and intravenous labetalol in hypertensive emergencies of pregnancy. Am J Obstet Gynecol.

[34] Raheem IA, Saaid R, Omar SZ, Tan PC (2012). Oral nifedipine versus intravenous labetalol for acute blood pressure control in hypertensive emergencies of pregnancy: a randomised trial. BJOG.

[35] Singhal S, Gupta AK (2015). A comparative randomized controlled parallel group study of efficacy and tolerability of labetalol versus methyldopa in the treatment of mild preeclampsia. Int J Basic Clin Pharmacol.

[36] Scardo JA, Vermillion ST, Newman RB, Chauhan SP, Hogg BB (1999). A randomized, double-blind, hemodynamic evaluation of nifedipine and labetalol in preeclamptic hypertensive emergencies. Am J Obstet Gynecol.

[37] Hall DR, Odendaal HJ, Steyn DW, Smith M (2000). Nifedipine or prazosin as a second agent to control early severe hypertension in pregnancy: a randomised controlled trial. BJOG.

[38] Aali BS, Nejad SS (2002). Nifedipine or hydralazine as a first-line agent to control hypertension in severe preeclampsia. Acta Obstet Gynecol Scand.

[39] Salama M, Rezk M, Gaber W, Hamza H, Marawan H, Gamal A, Abdallah S (2019). Methyldopa versus nifedipine or no medication for treatment of chronic hypertension during pregnancy: a multicenter randomized clinical trial. Pregnancy Hypertens.

[40] Cleary EM, Racchi NW, Patton KG, Kudrimoti M, Costantine MM, Rood KM (2023). Trial of intrapartum extended-release nifedipine to prevent severe hypertension among pregnant individuals with preeclampsia with severe features. Hypertension.

[41] Tita AT, Szychowski JM, Boggess K (2022). Treatment for mild chronic hypertension during pregnancy. N Engl J Med.

[42] Sanusi AA, Leach J, Boggess K (2024). Pregnancy outcomes of nifedipine compared with labetalol for oral treatment of mild chronic hypertension. Obstet Gynecol.

[43] Easterling T, Mundle S, Bracken H (2019). Oral antihypertensive regimens (nifedipine retard, labetalol, and methyldopa) for management of severe hypertension in pregnancy: an open-label, randomised controlled trial. Lancet.

[44] Chethy S (2024). A randomised parallel-group trial for comparison of safety and efficacy of oral nifedipine retard versus intravenous labetalol in management of hypertensive emergencies of pregnancy. Vijayapura, Karnataka: BLDE (Deemed.

[45] Kaur T, Kumari K, Rai P, Gupta V, Pandey S, Vineeta Vineeta, Saini S (2024). A comparative study of oral nifedipine and intravenous labetalol for acute hypertensive management in pregnancy: assessing feto-maternal outcomes in a hospital-based randomized control trial. Int J MCH AIDS.

[46] Nimbark N, Sharma R, Jain S (2024). Comparison of the efficacy of labetalol and nifedipine in preeclampsia: a prospective interventional study. J Clin Diagn Res.

[47] Jhaveri P, Shah D, Chauhan K (2024). Comparative efficacy and safety of oral labetalol and nifedipine in the management of preeclampsia: a randomized controlled trial. Int J Med Res.

[48] Rose DT, Jeyarani P (2019). Comparative study of labetalol and nifedipine in management of non-severe preeclampsia and its fetomaternal outcome. Int J Reprod Contracept Obstet Gynecol.

